# Prediction and analysis of three gene families related to leaf rust (*Puccinia triticina*) resistance in wheat (*Triticum aestivum* L.)

**DOI:** 10.1186/s12870-017-1056-9

**Published:** 2017-06-20

**Authors:** Fred Y Peng, Rong-Cai Yang

**Affiliations:** 1 Department of Agricultural, Food and Nutritional Science, University of Alberta, 410 Agriculture/Forestry Centre, Edmonton, AB T6G 2P5 Canada; 2 Feed Crops Section, Alberta Agriculture and Forestry, 7000 - 113 Street, Edmonton, AB T6H 5T6 Canada

**Keywords:** ABC transporter, NLR (NBS-LRR), START, Rust resistance genes, Molecular markers, Single nucleotide polymorphism (SNP), Bread wheat, *Triticum aestivum*, Genome analysis

## Abstract

**Background:**

The resistance to leaf rust (*Lr*) caused by *Puccinia triticina* in wheat (*Triticum aestivum* L.) has been well studied over the past decades with over 70 *Lr* genes being mapped on different chromosomes and numerous QTLs (quantitative trait loci) being detected or mapped using DNA markers. Such resistance is often divided into race-specific and race-nonspecific resistance. The race-nonspecific resistance can be further divided into resistance to most or all races of the same pathogen and resistance to multiple pathogens. At the molecular level, these three types of resistance may cover across the whole spectrum of pathogen specificities that are controlled by genes encoding different protein families in wheat. The objective of this study is to predict and analyze genes in three such families: NBS-LRR (nucleotide-binding sites and leucine-rich repeats or NLR), START (Steroidogenic Acute Regulatory protein [STaR] related lipid-transfer) and ABC (ATP-Binding Cassette) transporter. The focus of the analysis is on the patterns of relationships between these protein-coding genes within the gene families and QTLs detected for leaf rust resistance.

**Results:**

We predicted 526 *ABC*, 1117 *NLR* and 144 *START* genes in the hexaploid wheat genome through a domain analysis of wheat proteome. Of the 1809 SNPs from leaf rust resistance QTLs in seedling and adult stages of wheat, 126 SNPs were found within coding regions of these genes or their neighborhood (5 Kb upstream from transcription start site [TSS] or downstream from transcription termination site [TTS] of the genes). Forty-three of these SNPs for adult resistance and 18 SNPs for seedling resistance reside within coding or neighboring regions of the *ABC* genes whereas 14 SNPs for adult resistance and 29 SNPs for seedling resistance reside within coding or neighboring regions of the *NLR* gene. Moreover, we found 17 nonsynonymous SNPs for adult resistance and five SNPs for seedling resistance in the *ABC* genes, and five nonsynonymous SNPs for adult resistance and six SNPs for seedling resistance in the *NLR* genes. Most of these coding SNPs were predicted to alter encoded amino acids and such information may serve as a starting point towards more thorough molecular and functional characterization of the designated *Lr* genes. Using the primer sequences of 99 known non-SNP markers from leaf rust resistance QTLs, we found candidate genes closely linked to these markers, including *Lr34* with distances to its two gene-specific markers being 1212 bases (to c*ssfr1*) and 2189 bases (to *cssfr2*).

**Conclusion:**

This study represents a comprehensive analysis of *ABC*, *NLR* and *START* genes in the hexaploid wheat genome and their physical relationships with QTLs for leaf rust resistance at seedling and adult stages. Our analysis suggests that the *ABC* (and *START*) genes are more likely to be co-located with QTLs for race-nonspecific, adult resistance whereas the *NLR* genes are more likely to be co-located with QTLs for race-specific resistance that would be often expressed at the seedling stage. Though our analysis was hampered by inaccurate or unknown physical positions of numerous QTLs due to the incomplete assembly of the complex hexaploid wheat genome that is currently available, the observed associations between (i) QTLs for race-specific resistance and *NLR* genes and (ii) QTLs for nonspecific resistance and *ABC* genes will help discover SNP variants for leaf rust resistance at seedling and adult stages. The genes containing nonsynonymous SNPs are promising candidates that can be investigated in future studies as potential new sources of leaf rust resistance in wheat breeding.

**Electronic supplementary material:**

The online version of this article (doi:10.1186/s12870-017-1056-9) contains supplementary material, which is available to authorized users.

## Background

Leaf rust is a fungal disease caused by *Puccinia triticina* (= *P. recondita* Roberge ex Desmaz. f. sp. *tritici*.) which has been a serious threat to the world production of bread wheat (*Triticum aestivum* L.) and other cereals over the past decades [1–3]. The leaf rust pathogen is a biotrophic parasite with many physiological races that often are highly specific to wheat cultivars with compatible resistance genes. To date, at least 75 leaf rust (*Lr*) resistance genes have been identified, and the majority of them confer the race-specific resistance in the seedling stage [4]. However, a few race-nonspecific *Lr* genes including *Lr34* and *Lr67* have been also found particularly at adult stage, conferring resistance to multiple pathogen species [5–9].

According to Krattinger et al. [10], the *Lr* genes in wheat and other cereals may be divided into three groups based on their specificity and durability. The first group contains genes that confer race-specific resistance against one but not other races of the same pathogen species. As mentioned above, the majority of the *Lr* genes are within this group. These *Lr* genes often encode intracellular immune receptor proteins with nucleotide-binding sites and leucine-rich repeats (NLR, also known as NBS-LRR or *R* genes). Proteins encoded by *R* genes directly or indirectly perceive pathogen-derived virulence effectors that are secreted into the cytoplasm of host cells in order to suppress basal immunity. The second group contains genes that confer race-nonspecific resistance against multiple fungal pathogens simultaneously. The well-studied example in this group is *Lr34*. This locus was first reported by Dyck [11] and it was mapped on wheat chromosome 7D. It has been subsequently known to confer resistance against multiple diseases including leaf rust with the resistance gene named as *Lr34*, stem rust (caused by *P. graminis*) with the resistance gene named as *Sr57*, stripe (yellow) rust (caused by *P. striiformis*) with the resistance gene named as *Yr18*, powdery mildew (caused by *Blumeria graminis*) with the resistance gene named as *Pm38* and barley yellow dwarf virus with the resistance gene named as *Bdv*1 [11–14]. Thus, *Lr34* has other designations including *Lr34*/*Yr18, Lr34*/*Yr18/Sr57/Pm38* and *Lr34*/*Yr18/Sr57/Pm38/Bdv*1 in the literature. *Lr34* has been molecularly characterized and it encodes a putative ABC transporter containing transmembrane (TM) and nucleotide binding (NB) domains [6, 15]. The third group, like the second group, confers race-nonspecific resistance, but unlike the second group, such resistance is against all races within the same pathogen species. A known example in this group is a gene with resistance to stripe (yellow) rust in wheat (*Yr36*), and the resistance genes in the group are called START genes because they code for a START (steroidogenic acute regulatory [StAR] protein-related lipid transfer domain) protein [16]. Additional examples of *START* genes in other cereal species include the recessive rice blast resistance gene *pi21* encoding a small proline-rich protein [17], and the recessive barley powdery mildew resistance gene *mlo* coding for a membrane-anchored protein [18].

A common feature among the NLR, ABC and START proteins described above is the presence of distinct domains within each family. In the NLR group, a large number of potential R-genes or resistance gene analog (RGAs) encode R-proteins or effector-recognition receptors known as intracellular immune receptors and most belong to nucleotide-binding site-LRR (NBS-LRR or NLR) class [19] including seven domains or motifs: Toll/Interleukin-1 receptor (TIR-NBS-LRR or TNL), coiled-coil (CC- NBS-LRR or CNL), leucine zipper (LZ), NBS, LRR, TM and serinethreonine kinase (STK) [20]. In the ABC group, the only well-characterized gene (*Lr34*) encodes a full-size ATP-binding cassette (ABC) transporter and this protein is a member of the ABCG subfamily which is also known as the PDR (pleiotropic drug resistance) subfamily [6, 10, 12] and this protein impedes the invasion and spread of compatible pathogens in wheat and other cereals [21]. The functions of *Lr34* are constitutive rather than induced because the gene functions irrespective of whether or not pathogens are present. There is no known *Lr* gene that is yet available in the START group. *Yr36* is the only rust resistance gene in the group that is currently known to confer resistance to a broad spectrum of stripe rust races in wheat and this adult resistance is highly expressed at high temperatures (25-35 °C) [16]. In general, the *START* genes are not well studied but they are known to encode proteins for many functions in plants. The first function of the START proteins is, of course, the resistance to plant pathogens (e.g., the three START genes described above: *Yr36*, *pi21* [17] and *mlo* [18]). The second function is the response to abiotic stresses, e.g., increased expression of transmembrane START (TM-START) genes in chickpea in response to drought, salt, wound and heat stresses [22]. The third function is the ability to modulate transcription factor activity in Arabidopsis [23].

RNA-Seq is a more accurate method of quantifying gene expression levels than previous expression assay techniques such as microarray [24]. As RNA-Seq works without need for a genome sequence, it enables joint assays of host and pathogen transcriptomes, thereby gaining insights into how pathogens regulate the expression of their genes for disease progression and how they influence the host plant’s circuitry during a defense response [25–27]. A recent study [27] reported a detailed RNA-Seq time-course for a susceptible wheat cultivar (Vuka) and a resistant line (Avocet-*Yr5*) inoculated with the wheat yellow rust pathogen *Puccinia striiformis* f. sp. *tritici* (*Pst*) at different days post-inoculation (dpi). These authors were able to identify clusters of differentially expressed genes in wheat plants and *Pst*. For example, they identified a total of seven clusters of genes with similar expression profiles that were enriched in GO (gene ontology) term annotations and KEGG (Kyoto Encyclopedia of Genes and Genomes) pathway memberships for the wheat host. In particular, their Cluster III of host genes had a peak expression at 11 dpi and those genes for membrane transport and ABC transporters and chitinases in this cluster were significantly enriched. Thus, it would be desired to identify the expression profiles of the host genes that belong to the *ABC*, *NLR* and *START* gene families.

Recent QTL mapping studies have reported several hundred QTLs for rust resistance in wheat populations [28–31]. Additionally, novel QTLs associated with wheat rust resistance have been reported in several genome-wide association studies (GWAS) [32–34], using the 9 K or 90 K single nucleotide polymorphism (SNP) chips [35, 36]. Most of the QTLs for leaf rust resistance at the seedling and adult stages are now stored in the T3 database [37]. However, little is known about the physical relationships between these QTLs and the *ABC*, *NLR* and *START* genes. For example, it may be expected [10] that the *ABC* (and *START*) genes are more likely to share genomic regions with QTLs for race-nonspecific adult resistance whereas the *NLR* genes are more likely to share genomic regions with QTLs for race-specific resistance that is readily expressed in the seedlings. The objective of this study is to conduct bioinformatic prediction and gene annotation within *ABC*, *NLR* and *START* gene families in wheat that share genomic regions with QTLs for leaf rust resistance at the seedling and adult stages as obtained from the T3 database [37]. Specifically, we first predicted the putative *ABC*, *NLR* and *START* genes across the wheat genome. Then we attempted to establish physical relationships between these putative genes and the designated *Lr* genes from the sequences of SNP markers flanking the leaf rust QTLs. Together, this work provides an important framework for future studies to discover molecular functions of existing and new rust resistance genes in the wheat gene pool for their deployment in the development of wheat cultivars with improved rust resistance.

## Results

### Putative ABC, NLR and START proteins in wheat

In total, we predicted 526 *ABC* genes in the wheat genome. These ABC proteins were classified into eight subfamilies with the subfamily G further divided into Gwbc and Gpdr (Table 1). Our results show that the G (Gwbc and Gpdr), C, B and I subfamilies in wheat are the four largest in the ABC family, accounting for 30.4%, 23.0%, 18.8% and 16.2% of total 526 *ABC* genes, respectively. Notably, 78 ABC Gpdr transporters were predicted in the wheat genome, slightly more than a previous estimate of 60 full-size ABCG transporters estimated from the full-size ABCG gene numbers in Arabidopsis and rice [15, 38]. In comparison, we also performed a parallel analysis in Arabidopsis to validate the prediction pipeline. Our result showed that, of 131 ABC proteins found in Arabidopsis G (33.5%), B (20.8%), I (16.1%), C (12.8%) were the four largest subfamilies, which is consistent with the Arabidopsis result on the ABC proteins recently reported by Andolfo et al. [39]. These authors also reported 146 ABC proteins in rice [39], including 7 (4.8%), 29 (19.9%), 18 (12.3%), 3 (2.1%), 3 (2.1%), 7 (4.8%), 41 (28.1%), 22 (15.1%), and 16 (11.0%) in A, B, C, D, E, F, Gwbc, Gpdr and I families, respectively.

**Table 1:** Numbers of ABC, NLR and START family proteins and their subfamily classes over three wheat genomes (A, B and D) and chi-squared tests for even distributions across the genomes

		Wheat genome					
Protein family	Class	A	B	D	Total (%)	χ^2^	*P*-value
ABC	A	6	6	7	19 (3.6)	0.105	0.949
	B	31	36	32	99 (18.8)	0.424	0.809
	C	32	49	40	121 (23.0)	3.587	0.166
	D	5	4	4	13 (2.5)	0.154	0.926
	E	3	4	4	11 (2.1)	0.182	0.913
	F	6	6	6	18 (3.4)	0.000	1.000
	Gpdr	24	28	26	82 (15.6)	0.308	0.857
	Gwbc	28	30	24	78 (14.8)	0.683	0.711
	I	30	26	29	85 (16.2)	0.306	0.858
	Total	165	189	172	526	1.738	0.419
NLR	CNL	320	346	313	979 (87.6)	1.853	0.396
	TNL	3	3	3	9 (0.8)	0.000	1.000
	N/A	47	46	36	129 (11.6)	1.721	0.423
	Total	370	395	352	1117	2.505	0.286
START	HD-ST	3	4	4	47 (32.6)	0.182	0.913
	HD-ST	2	2	3	54 (37.5)	0.286	0.867
	MINIM	16	16	15	11 (7.6)	0.043	0.979
	START	15	20	19	7 (4.9)	0.778	0.678
	OTHER	9	9	7	25 (17.4)	0.320	0.852
	Total	45	51	48	144	0.375	0.829

*Abbreviations*: *ABC* ATP-binding cassette; *NLR* nucleotide-binding site /leucine-rich repeat (NBS-LRR); *START* steroidogenic acute regulatory (StAR) protein-related lipid transfer domain

For the NLR family, we predicted 1117 NLR proteins in wheat (Table 1). This number is slightly higher than those in previous studies of the genome-wide analysis of NLR-coding genes in wheat [40, 41] but slightly lower than the 1185 NLR proteins predicted using the new gene models in the wheat genome assembly recently generated by Earlham Institute [42]. In a concurrent analysis, we predicted 249 NLR proteins in Arabidopsis and the majority of them were TNL (Tir-NBS-LRR), accounting for nearly 69% (171), compared to less than 21% (52) of CNL (Coiled-coil-NBS-LRR) proteins. This high TNL/CNL ratio was consistent with previous studies of the NLR proteins in dicot species [40, 43, 44]. In contrast, nearly 88% (979) of the wheat NLR proteins were classified to the CNL class. However, nine (0.8%) wheat TNL proteins were detected using NLR-Parser, contrary to the commonly accepted belief that TNL genes might have been lost completely in monocots [19, 40, 45]. These nine proteins were further analyzed with PfamScan [46] and the analysis indicated that none of them contains a predicted TIR domain (PF01582), The analysis also showed that eight of them contain a truncated NB-ARC domain (PF00931) whose length ranges from 63 to 271 amino acids (the full length of the NB-ARC domain is 288 in Pfam) and the remaining one (Traes_3B_706A56165) was annotated by PfamScan as a Ceramidase family protein. Thus, this limited number of TNL proteins predicted in wheat is likely due to NLR-Parser annotation error arising from the presence of truncated NB-ARC domains. In addition, 10.4% in Arabidopsis and 11.6% in wheat of the NLR proteins could not be classified to either CNL or TNL, which were designated as ‘N/A’ by NLR-Parser.

The *START* family genes were predicted exclusively based on the presence of START (IPR002913 and IPR005031) and/or START-like domain (IPR023393) following domain analysis of the wheat proteome using InterProscan [47]. From this analysis, we detected 139 and 144 *START* genes in Arabidopsis and wheat, respectively. Among the five classes, the START class was the largest in both Arabidopsis and wheat, with 75 (54%) in the former and 54 (37.5%) in the latter. HD-START was the second largest class in Arabidopsis with 21 members (15.1%), whereas MINIMAL-START was the second largest class in wheat with 47 members (32.6%). In both species, HD-START-MEKHLA was the smallest, each with seven members accounting for 5.0% in Arabidopsis and 4.9% in wheat. In an early study, only 35 Arabidopsis *START* genes were reported [48], but these authors used only five START-domain proteins (Arabidopsis GL2 and ATML1, rice ROC1 and AAP54082, and human PCTP) as queries for BLAST searches. Recently, Satheeth and colleagues [22] reported 36 *START* genes in chickpea (*Cicer arietinum* L.), using the START domain (~200 amino acids) encoded by the Arabidopsis gene *AT5G54170* (in our Arabidopsis *START* gene list) as query sequence in the TBLASTN search against the genome contigs database of chickpea. To the best of our knowledge, no genome-scale analysis of START proteins has been reported in wheat.

**Fig. 1:**
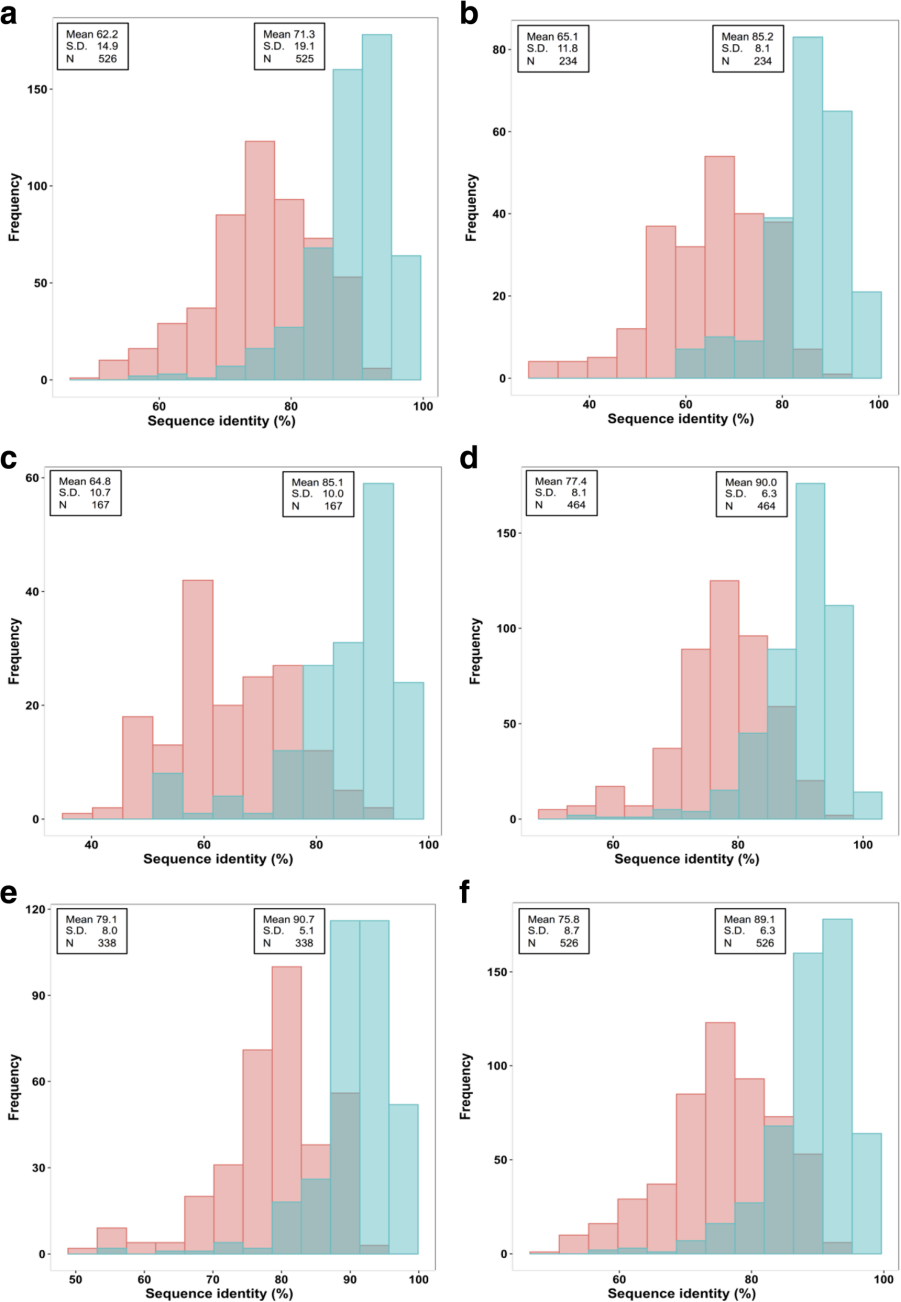
The sequence percent identity between wheat ABC proteins and their critical domains and those in Arabidopsis and *Oryza sativa* (rice). **a**: entire ABC protein sequences; **b**: ABC transporter type 1 transmembrane domain (IPR011527); **c**: ABC-2 transporter domain (IPR013525); **d**: ABC transporter-like (IPR003439); **e**: AAA+ ATPase domain (IPR003539); **f**: P-loop NTPase fold (IPR027417). *Red*: wheat-Arabidopsis comparison; *green*: wheat-rice comparsion; *grey green*: overlapping tails of the two comparisons. The average sequence identity (Mean), standard deviation (S.D.) and number of comparisons (N) are indicated in the two text boxes: wheat-Arabidopsis at the left side and wheat-rice comparison at the right side

Our prediction was primarily based on domain analysis, with their domains summarized in Additional file 1. However, our classification of the putative ABC proteins in wheat into different subfamilies was based on protein sequence homology to the ABCs in Arabidopsis and rice (*Oryza sativa*). Therefore, we analyzed sequence similarity of ABC proteins and their two main types of domains (transmembrane domains or TMDs, and cytosolic nucleotide binding domains or NBDs) between wheat, Arabidopsis and rice (Fig. 1). The protein sequence identity was higher between wheat and rice than between wheat and Arabidopsis. On average, these entire ABC proteins shared 62.2% sequence identity (S.D. ± 14.9%) between wheat and Arabidopsis, and 71.3% (S.D. ± 19.1%) between wheat and rice (Fig. 1a). As expected, the ABC domains showed greater homology in both wheat-Arabidopsis and wheat-rice comparisons, especially for the NBDs including ABC transporter-like domain (IPR003439), AAA+ ATPase domain (IPR003593) and P-loop NTPase fold (IPR027417). For instance, the ABC transporter type 1 transmembrane domain (IPR011527) had an average sequence identity of 65.1% between wheat and Arabidopsis, and 85.2% between wheat and rice (Fig. 1b). Likewise, the average identity of the ABC-2 transporter domain (IPR013525) was 64.8% between wheat and Arabidopsis, and 85.1% between wheat and rice (Fig. 1c). For the ABC transporter-like domain (IPR003439) the average identity increased to 77.4% between wheat and Arabidopsis, and 90.0% between wheat and rice (Fig. 1d). The homology of the AAA+ ATPase domain (IPR003593) was even higher between these species, with the average identity reaching 79.1% between wheat and Arabidopsis and 90.7% between wheat and rice (Fig. 1e). Lastly, the P-loop NTPase fold (IPR027417) showed slightly lower homology, with an average idenitity of 75.8% between wheat and Arabidopsis and 89.1% between wheat and rice (Fig. 1f). Although the sequence identity between wheat and rice ABC proteins is mostly higher than between wheat and Arabidopsis, the top hit of some wheat ABC proteins was from Arabidopsis. Thus, we used both Arabidopsis and rice ABCs to classfiy the wheat ABC proteins.

### Annotation of the wheat *ABC*, *NLR* and *START* genes

**Fig. 2:**
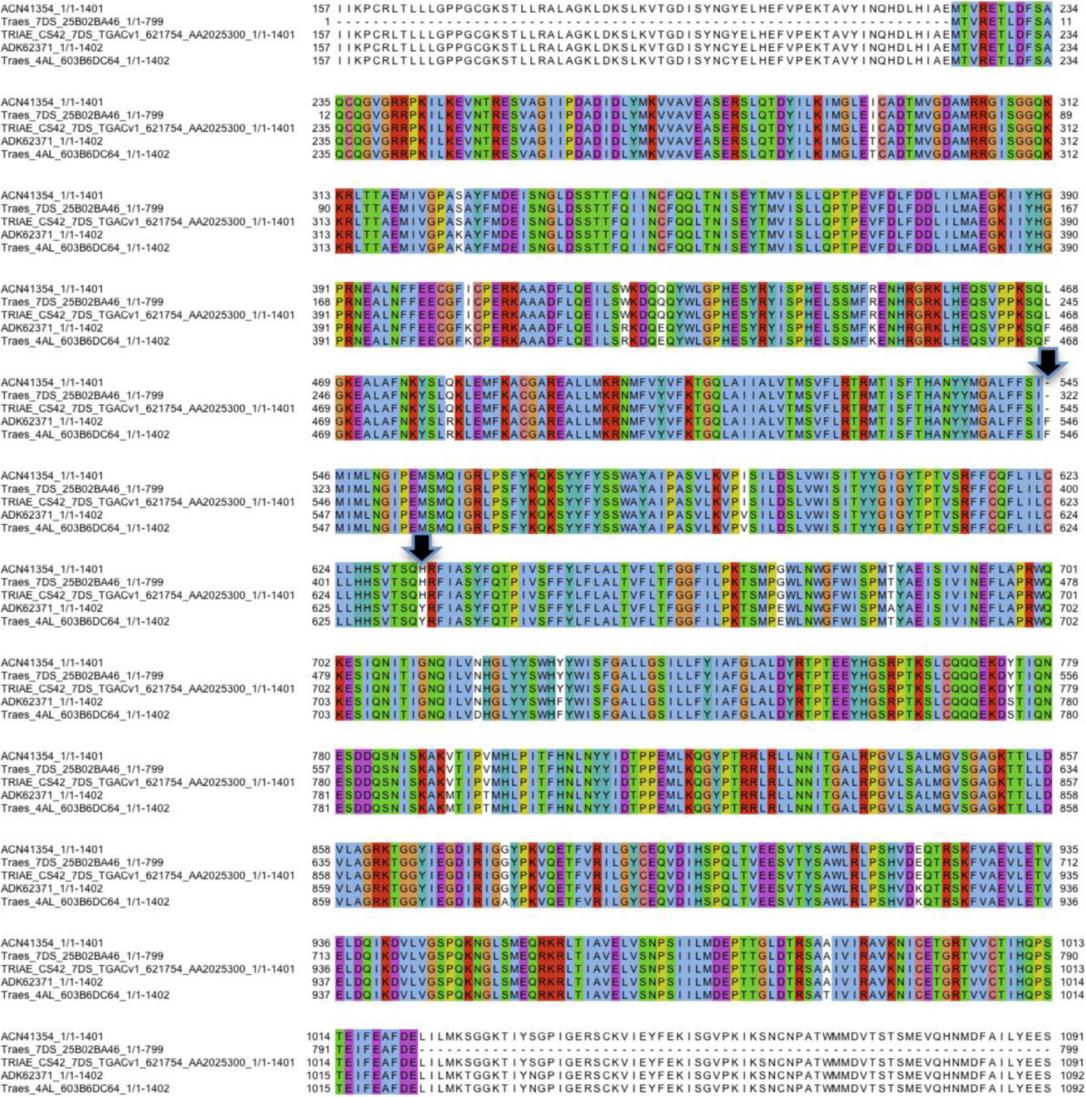
Protein sequence alignment of the resistant and susceptible Lr34-D in Genbank and putative Lr34-D and Lr34-B in Ensembl. ACN41354: resistance version of GenBank LR34-D; ADK62371: susceptibility version of GenBank LR34-D; Traes_7DS_25B02BA46: LR34-D in Ensembl release 31 (partial); TRIAE_CS42_7DS_TGACv1_621754_AA2025300: LR34-D in the Ensembl release 33 (full length). Traes_4AL_603B6DC64: LR34-B in Ensembl release 31. The protein length is shown after the slash (‘/’) of each identifier. *Arrows* indicate the deletion of the phenylalanine residue (Phe/F) on position 546 and tyrosine (Y) substitution to histidine (His/H) on position 634 in LR34res-D. The beginning and ending regions of the sequence alignment were removed for clarity

As almost all *ABC*, *NLR* and *START* genes have no informative annotation (only ambiguous annotation such as uncharacterized protein or hypothetical protein) in the Ensembl Plants database [49], we enhanced their annotation using sequence analysis with BLAST [50]. We first performed BLAST searches against the known plant disease resistance genes in GenBank and PRGdb [51, 52]. Though 145 ABC proteins showed various degrees of sequence similarity to the known LR34 protein, only two had more than 97% identity (Additional file 2), suggesting that the respective *Lr34* and *Lr34-B* genes are *Traes_7DS_25B02BA46* and *Traes_4AL_603B6DC64* in Ensembl. *Traes_7DS_25B02BA46* is partial, encoding only 799 amino acids (aa) instead of 1401 aa for the full-length LR34 (Additional file 2). In the Ensembl release 33, we found the full-length *Lr34* gene (TRIAE_CS42_7DS_TGACv1_621754_AA2025300) encoding a protein of 1401 aa. Our annotation of *Lr34* and *Lr34-B* was confirmed by multiple sequence alignment (MSA) of the LR34 and LR34-B proteins in Ensembl as well as the known resistant (accession: ACN41354) and susceptible (ADK62371) LR34 proteins in GenBank (Fig. 2). Interestingly, this MSA indicated that the putative LR34 proteins in Ensembl (both Traes_7DS_25B02BA46 in release 31 and TRIAE_CS42_7DS_TGACv1_621754_AA2025300 in release 33) represent the resistant version of LR34. In addition, the putative LR34-B (Traes_4AL_603B6DC64) protein has about 97% sequence identity with LR34 (Additional file 2), consistent with a previous study [15]. In contrast, in the NLR and START families, no genes showed over 90% sequence identity with known rust related proteins, and thus unlikely to represent the same genes. For example, Traes_1DS_5FF8D9E2D in the NLR family exhibited an identity of ~85% with the known stripe rust resistance protein YR10 in GenBank (accession: AAG42168). In the START family, only the sequence of a kinase-START domain protein from *T. dicoccoides* was found in GenBank (ACF33195), and the highest identity was ~63% at the protein sequence level with Traes_7DL_A330C0F90 (Additional file 2). The relative low sequence identity may be attributed to (i) the use of Chinese Spring (CS) for genome sequencing and different wheat cultivars for gene isolation and (ii) increased allelic diversity of *R* genes among the wheat accessions. For example, *Lr1* was cloned from Glenlea [7] and *Lr10* from Thatcher*Lr10* [8]. In contrast, it is reasonable to expect that when the same wheat genotype is used for both genome sequencing and gene cloning perfect sequence matching may be found. The only example we have found so far for this case is *Lr34* whose sequencing and cloning was based on CS [6] and its protein sequence showed 100% identity with putative LR34 we annotated in this study (Fig. 2; Additional file 2).

Since numerous Arabidopsis genes have been characterized, especially for the ABC family, of which the major functions were largely known [21, 38, 53–55] (summarized in Additional file 3: Table S1), we then annotated these genes based on their best hit in the Arabidopsis genome (Additional file 2). For example, the putative LR34 in Ensmebl was annotated as ATPDR5 (Pleiotropic Drug Resistance 5, AT2G37280, also known as ABCG33 or ABC transporter G family member 33). Similarly, LR34-B (Traes_4AL_603B6DC64) was annotated as ATPDR9 (Pleiotropic Drug Resistance 9, AT3G53480, also known as ABCG37 or ABC transporter G family member 37). Thus, this annotation can be used to help select candidate genes for molecular characterization in wheat by leveraging the knowledge in the model plant.

### Distribution of *ABC*, *NLR* and *START* genes in the three genomes of wheat and their physical proximity to SNPs of QTLs for leaf rust resistance

Of the *ABC* genes we predicted in this study, 165, 189 and 172 are from the A, B, and D genomes, respectively (Table 1). For *NLR* genes, 370 (CNL: 320, TNL: 3, N/A: 47), 395 (CNL: 346, TNL: 3, N/A: 46) and 352 (CNL: 313, TNL: 3, N/A: 36) are from A, B and D genomes respectively. For the *START* family: 45 (START: 15, HD-START: 3, HD-START-MEKHLA: 2, MINIMAL-START: 16, OTHER: 9), 51 (START: 20, HD-START: 4, HD-START-MEKHLA: 2, MINIMAL-START: 16, OTHER: 9), and 48 (START: 19, HD-START: 4, HD-START-MEKHLA: 3, MINIMAL-START: 15, OTHER: 7) are from A, B and D genomes respectively. The *ABC*, *NLR* and *START* genes were evenly distributed over the three wheat genomes (A, B and D), judging from insignificant chi-squared (χ^2^) tests for individual subfamily classes.

From the two data sets each with 2500 leaf rust resistance QTLs being downloaded from the T3 database [37], we retained 1090 and 1542 significant (*P* ≤ 0.01) QTLs for seedling and adult resistance, respectively. In these two data sets, a total of 1809 unique SNP markers were used (Additional file 4), and 451 of them are present in the QTLs of both seedlings and adult plants (Additional file 3: Fig. S1A). Because a total of 570 SNPs for seedling and adult resistance (representing 391 unique markers) have unknown (UNK) chromosomes (Additional file 3: Fig. S1B and C), we mapped their flanking sequences to the wheat genome to determine if these SNPs are located within the ABC, NLR and START genes analyzed in this study or their neighboring regions (5 Kb upstream of 5′ transcription start site [TSS] or downstream of 3′ transcription termination site [TTS] to the predicted genes).

**Fig. 3:**
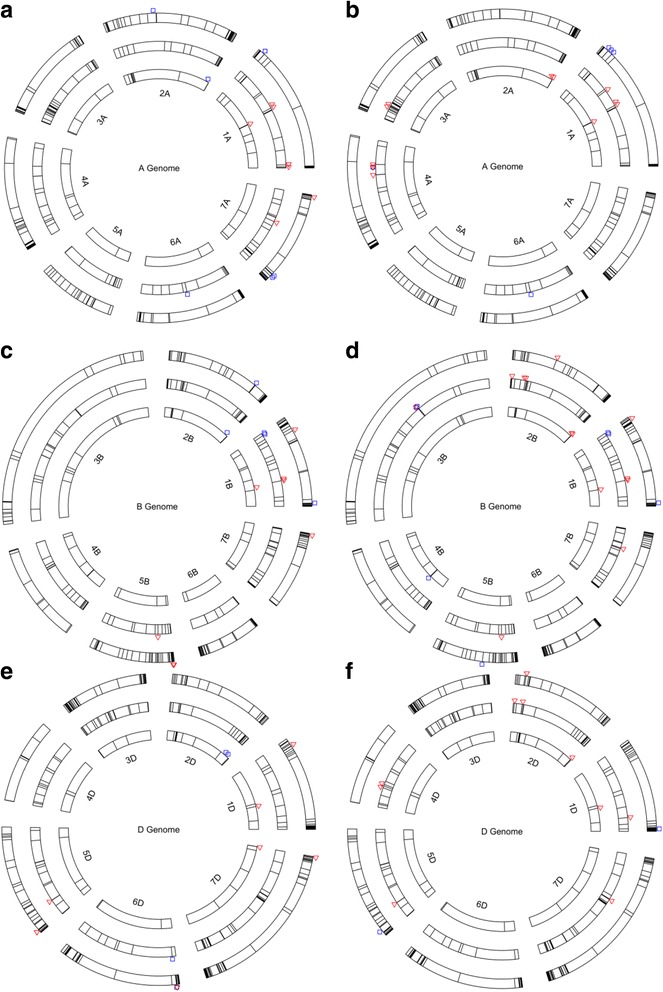
Distribution of *ABC*, *NLR* and *START* genes and SNP markers for leaf rust resistance in the A, B and D genomes of the hexaploid wheat genome. The circles were arranged according to the size of each family: inner, middle and outer circles for *START*, *ABC* and *NLR* genes. **a**: seedling A genome; **b**: adult A genome; **c**: seedling B genome **d**: adult B genome; **e**: seedling D genome; **f**: adult D genome. *Red* △ denotes markers within a gene; *Blue* □ denotes markers 5 Kb upstream (from the transcription start site or TSS) or downstream (from the transcription termination site or TTS) of a gene. Seven SNPs overlapped because of their close physical proximity on the same chromosome, see Additional file 5 for details. We do not show designated leaf rust (*Lr*) genes on the chromosome maps for seedling resistance and adult plant resistance, which are listed as follows: 1A: *Lr59*; 2A: *Lr11*, *Lr17a*, *Lr17b*, *Lr37*, *Lr38*, *Lr45*, *Lr49*; 3A: *Lr63*; 4A: *Lr28*; 5A: -; 6A: *Lr56*, *Lr62*, *Lr64*; 7A: *Lr20*, *Lr47*; 1B: *Lr33*, *Lr44*, *Lr46*, Lr51, Lr55; 2B: Lr13, Lr16, Lr23, Lr35, Lr50, Lr58; 3B: Lr27; 4B: Lr12, Lr25, Lr30, *Lr31*, *Lr48*; 5B: *Lr18*, *Lr52*; 6B: *Lr3a*, *Lr3bg*, *Lr3ka*, *Lr9*, *Lr36*, *Lr53*, *Lr61*; 7B: *Lr14a*, *Lr14b*; 1D: *Lr21*, *Lr42*, *Lr60*; 2D: *Lr2a*, *Lr2b*, *Lr2c*, *Lr15*, *Lr22a*, *Lr22b*, *Lr39*, *Lr54*; 3D: *Lr24*, *Lr32*; 4D: -; 5D: *Lr1*, *Lr57*; 6D: -; 7D: *Lr19*, *Lr29*, *Lr34*

The predicted *ABC*, *NLR* and *START* genes and the SNPs for seedling and adult resistance that reside within coding or neighboring (5 Kb upstream or downstream) regions of the predicted genes were mapped onto individual chromosomes in each of the three wheat genomes (A, B and D) (Fig. 3). The designated *Lr* genes previously mapped on each chromosome are also shown in the outer layer in Fig. 3 to indicate their physical affinity with the candidate genes and SNPs for leaf rust resistance. Unfortunately such affinity is very coarse due to the lack of mapping positions of the *Lr* genes on individual chromosomes. Some of the candidate genes with leaf rust SNPs only have positions on a scaffold (a set of ordered sequence contigs with gaps between them), and thus could not be depicted in Fig. 3 (Additional file 5). For this reason, we divided these SNPs into two categories: the genes with chromosome positions (WCP) and the genes with scaffold positions (WSP). The numbers of SNPs for seedling and adult resistance residing in coding regions of *ABC*, *NLR* and *START* genes or their neighborhood (within or 5 Kb upstream of 5′ end or downstream of 3′ end) showed different patterns (Table 2). For example, we found 18 SNPs in genic or neighboring regions of the *ABC* genes in seedlings and 39 in adult plants. Conversely, 29 and 14 SNPs for seedling and adult resistance were found in genic or neighboring regions of the *NLR* genes, respectively. In contrast, the START genes or their neighborhood contain roughly identical numbers of QTL SNPs for both seedlings and adult resistance.

**Table 2:** Numbers of SNPs that reside within the *ABC*, *NLR* and *START* genes or their neighborhoods^a^

	Seedling			Adult		
	WCP^b^	WSP	Total	WCP	WSP	Total
ABC	14 (9)^c^	4 (3)	18 (12)	31 (19)	12 (8)	43 (27)
NLR	21 (15)	8 (6)	29 (21)	11 (8)	3 (2)	14 (10)
START	9 (7)	3 (3)	12 (10)	9 (7)	1 (1)	10 (8)

These SNPs are the markers of QTLs identified for leaf rust resistance in wheat seedlings and adult plants

^a^Neighborhood defined as 5 Kb upstream (from 5′ transcription start site or TSS) or downstream (from 3′ transcription termination site or TTS) of a gene, see Additional file 5 for details

^b^WCP, With known Chromosome Positions; WSP, With only Scaffold Positions

^c^The number of involved genes is indicated in parentheses

Excluding the SNPs in non-coding and neighboring regions of *ABC* and *NLR* genes as shown in Table 2, we obtained a subset of SNPs that reside within coding regions and cause amino acid changes in these gene families (Table 3). These coding SNPs are the markers of QTLs identified for leaf rust resistance in wheat seedlings and adult plants as described above. The numbers of nonsynonymous SNPs in the coding regions of *ABC* and *NLR* genes exhibited different patterns in seedlings and adult plants. In the *ABC* genes, we found 17 missense SNPs for adult resistance and only five SNPs for seedling resistance. In contrast, in the *NLR* genes, we only predicted five nonsynonymous SNPs for adult resistance and six SNPs for seedling resistance (Table 3; Additional file 6). These candidate genes with nonsynonymous SNPs from the QTL data can be used in further studies to investigate their functional roles in *Lr* genes in wheat.

**Table 3:** Numbers of SNPs that reside within coding regions and cause amino acid changes in the ABC, NLR and START genes. These SNPs are the markers of QTLs identified for leaf rust resistance in wheat seedlings and adult plants^a^

	Seedling			Adult		
	WCP^b^	WSP	Total	WCP	WSP	Total
ABC	3 (3)^c^	2 (2)	5 (5)	10 (9)	7 (6)	17 (15)
NLR	4 (4)	2 (2)	6 (6)	3 (3)	2 (2)	5 (5)
START	1 (1)	0 (0)	1 (1)	2 (2)	1 (1)	3 (3)

^a^Seven synonymous SNPs (highlighted in Additional file 6) and their genes not included

^b^WCP, With known Chromosome Positions; WSP, With Scaffold Positions

^c^The number of involved genes is indicated in parentheses

### *ABC*, *NLR* and *START* genes linked to non-SNP molecular markers of rust resistance

The primer sequences of 99 non-SNP molecular markers linked to designated rust genes were used to identify corresponding candidate *ABC*, *NLR* and *START* genes in wheat. In total, we found 75 combinations of rust and Ensembl genes located near these markers, with the median physical distance of about 1.7 Mb (Additional file 7). Of these, eight genes are located less than 100 Kb away from the rust-associated markers, including two, five and one gene(s) in the *ABC*, *NLR* and *START* family, respectively (Table 4). For example, we found the *gwm350* marker is only 293 bp to *Traes_4AL_7AFBA6334*. This gene might therefore be *SrND643*, a temporarily designated stem rust gene conferring resistance to several Ug99 races of *Pgt* [56]. In addition, *Lr34* (*Yr18/Bdv1/Pm38/Ltn1*) on 7DS was associated with *Traes_7DS_25B02BA46*, with a distance of 1212 bp to *cssfr1* and 2189 bp to *cssfr2*, two known markers for *Lr34* [13, 57]. Along with Fig. 2 (above-mentioned MSA), this result further supports the annotation of *Traes_7DS_25B02BA46* as *Lr34*, demonstrating the usefulness of bioinformatics analyses in the discovery of rust resistance genes for functional characterization.

**Table 4:** Candidate genes less than 100 Kb to rust resistance markers for designated rust genes in the ABC, NLR and START families

Designated rust gene	Chromosome	Marker	Ensembl gene^a^	Family^b^	Distance^c^
*SrND643*	4AL	*gwm350*	4AL_7AFBA6334	NLR (CNL)	293
*Lr37/Sr38/Yr17*	2AS	*Ventriup-LN2*	2AS_71B82606A	START (START)	503
*Sr26*	6AL	*BE518379*	6AL_91A76CBCC	ABC (I)	561
*Lr34/Yr18/Bdv1/Pm38/Ltn1*	7DS	*cssfr1*	7DS_25B02BA46^d^	ABC (Gpdr)	1212
*Lr34/Yr18/Bdv1/Pm38/Ltn1*	7DS	*cssfr2*	7DS_25B02BA46^d^	ABC (Gpdr)	2189
*Sr33*	1DS	*Xcfd15*	1DS_9EABB4B7A	NLR (CNL)	9523
*Lr46/Yr29/Pm39/Ltn*	1BL	*Xwmc719*	1BL_33D479058	NLR (CNL)	28,712
*Sr13*	6AL	*CD926040*	6AL_22F03E292	NLR (CNL)	29,257
*SrND643*	4AL	*wmc219*	4AL_7E627E983	NLR (CNL)	99,528

^a^The ‘Traes_’ prefix in each gene identifier removed

^b^Abbreviations: ABC, ATP-binding cassette; NLR, nucleotide-binding site /leucine-rich repeat (NB-LRR or NBS-LRR); START, steroidogenic acute regulatory (StAR) protein-related lipid transfer domain. The subgroup names were added in the parentheses. CNL: coiled-coil NBS-LRR; Gpdr: pleiotrpoic drug resitance class in G subfamily

^c^Physical distance in the number of bases between the gene start (5′ transcription start site or TSS) and marker end (marker located upstream of a gene) or the gene end (3′ transcription termination site or TTS) and marker start (marker located downstream of a gene)

^d^Traes_7DS_25B02BA46 represents the cloned *Lr34* gene (cf. Figure 2)

### Gene expression

**Fig. 4:**
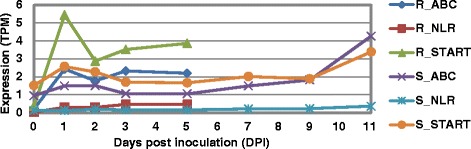
RNA-seq expression profiles of *ABC*, *NLR* and *START* genes in resistant (R) wheat line (Avocet-*Yr*5) and susceptible (S) wheat cultivar (Vuka) inoculated with *yellow* rust pathogen *Puccinia striiformis* f. sp. *tritici* (*Pst*) during 5 and 11 days post inoculation, respectively. Abbreviations: DPI, days post inoculation; TPM, number of transcripts per million reads; R_ABC, *ABC* gene expression in resistant wheat; R_NLR, *NLR* gene expression in resistant wheat; R_START, *START* gene expression in resistant wheat; S_ABC, *ABC* gene expression in susceptible wheat; S_NLR, *NLR* gene expression in susceptible wheat; S_START, *START* gene expression in susceptible wheat

The expression differences of the putative *ABC*, *NLR* and *START* genes at different time points of infection (days post inoculation or dpi) in resistant and susceptible wheat plants are presented in Additional file 8. Our analysis revealed that the expression levels of the *NLR* genes for both resistant and susceptible plants were close to zero across all time points with the resistant plants being expressed only slightly more that the susceptible plants at comparable dpi (Fig. 4). In contrast, we observed higher levels of gene expression in the resistant wheat plants than in susceptible for ABC and START genes at early stages of infections (≤ 5 dpi) but susceptible plants had the similar amount of expression at the later stage (11 dpi). This is consistent with the finding in Fig. 3 of Dobon et al. [27] in which seven clusters of host genes were identified and one cluster (Cluster III) including the genes encoding ABC transporters had a peak expression at 11 dpi.

However, this result needs to be interpreted with care. Resistant and susceptible plants may have very different genetic backgrounds (i.e. with other rust resistance genes than *Yr5* across the wheat genome) because they are different cultivars but not isogenic lines. Similar gene expression analyses were performed in the wheat cultivars Thatcher (susceptible) and its near-isogenic line (NIL) carrying a leaf rust resistance gene *Lr9* (Thatcher*Lr9*) or *Lr10* (Thatcher*Lr10*) (resistant) [58, 59], but both using an EST (expressed sequence tag) based approach. These analyses showed some genes were expressed at higher levels in the resistant cultivar, including an *ABC* transporter gene, while several *NLR* genes were expressed in both the susceptible and resistant samples infected with *Ptr*. Additionally, gene expression changes induced by the rust resistance gene *Lr34/Yr18* were reported in the wheat NILs using Affymetrix wheat GeneChip microarray [60], which identified both upregulated and downregulated genes associated with resistance.

## Discussion

The present study employed a domain analysis of wheat proteome to predict a total of 1787 genes, with 526 coding for ABC proteins, 1117 for NLR proteins and 144 for START proteins over the three wheat genomes (A, B and D) (Table 1, Fig. 3). We were able to identify a number of SNPs that reside within protein-coding regions and cause amino acid changes of the predicted *ABC*, *NLR* and *START* genes or their neighborhood (5 Kb upstream from 5′ end or downstream from 3′ end). A total of 59 such SNPs were identified as the markers of QTLs for leaf rust resistance at the seedling stage and another 67 SNPs as the markers of QTLs for leaf rust resistance at the adult stage (Table 2; Fig. 3). The number of adult-resistance SNPs residing in the *ABC* genes or their neighborhood at the adult stage was more than doubled the number of seedling-resistance SNPs whereas the reverse pattern was observed for the SNP variants associated with the *NLR* genes (Table 2). This seems consistent with the expectation [10] that the QTLs for race-nonspecific, adult rust resistance are more likely to share genomic regions with the candidate genes in the *ABC* (and *START*) family whereas those for race-specific rust resistance in the seedlings are more likely to share genomic regions with the candidate genes in the *NLR* family.

This study represents a major step towards classification of the candidate genes for race-specific and race-nonspecific resistance in the three gene families and their chromosome-wise physical affinity with SNPs (and thus QTLs) for leaf rust resistance in wheat. Such information will be valuable for further characterizing QTLs at molecular level and their functional relationships with the *ABC*, *NLR* and *START* genes. We were already able to document our predicted amino acid changes due to alternation of single SNPs (point mutation) in the coding regions of the *ABC*, *NLR* and *START* genes (Fig. 2; Table 3). Our analysis was motivated by recent reports that changes in two amino acids caused the difference in proteins encoded by the resistance and susceptibility alleles of *Lr*34 [6, 15] and *Lr*67 [61]. Nevertheless, our prediction based on point mutation must be treated as a preliminary result as structural variations such as copy number variation (CNV) of resistance genes may possess a more dramatic impact on disease and other biotic stress [62]. Further complication arises from temporal variation among expressions of the putative *ABC*, *NLR* and *START* genes in compatible (susceptible) and incompatible (resistant) wheat-rust interactions as evident in our RNA-Seq analysis (Fig. 4). Thus, the predictions based on point mutation such as ours can only serve as a starting point towards more thorough molecular and functional characterization of resistance genes for their eventual cloning.

Our prediction indicates that the number of race-specific *NLR* genes (*R* genes) is almost doubled the number of race-nonspecific genes (*ABC* and *START* genes). This seems consistent with the phenomenon that the majority of *Lr* genes identified so far in wheat are effective against single races of lest rust only. As Krattinger et al. [10] pointed out, this phenomenon is also true for resistances to other diseases in wheat and other cereals. The problem with the use of such race-specific *Lr* genes deployed in the wheat cultivars is that they have quickly become ineffective when new, more virulent races appear in leaf rust pathogen [63]. For this reason, wheat breeders and pathologists have focused on discovery, characterization and use of race-nonspecific genes for durable resistance. However, decades of genetic and breeding research have only been able to identify a limited number of genes in wheat with durable and broad-spectrum resistance to rusts including *Lr34/Yr18/Sr57/Pm38*, *Lr46/Yr29/Pm39*, *Lr67/Yr46/Sr55/Pm46*, *Lr68*, *Lr75,* and *Yr36* [5, 6, 15, 16, 61, 64, 65]. Thus far, *Lr34/Yr18/Sr57/Pm38* (or simply *Lr34* hereafter) is the most well-characterized multi-pathogen resistance gene located on wheat chromosome 7D [11]. Unfortunately we did not find any known SNPs for the leaf rust resistance in the T3 data sets [34] that would reside within coding regions of the *Lr34* gene or its neighborhood within 5 Kb upstream and downstream. Certainly, the wheat 9 K and 90 K SNP chips are hardly sufficient to cover large, complex wheat genomes, particularly the D genome (cf. Fig. 3). With ongoing international efforts on manifold sequencing of wheat genomes, exact physical positions and functions of those SNP variants within the *ABC* genes (Table 2) will be further clarified and characterized.

It may be questioned why *Lr67/Yr46/Sr55/Pm46* (or simply *Lr67*) was not included in this study as it is another well-characterized race-nonspecific gene at the molecular level just like *Lr34*. The reason is that it does not encode an ABC transporter, but rather it encodes a hexose transporter and confers a similar, but somewhat reduced partial resistance to all three wheat rust pathogens and powdery mildew [62]. Like in *Lr34*, only two amino acid substitutions (Arg144Gly and Leu387Val) distinguish the resistance (LR67res) from susceptibility (LR67sus) protein [62]. The hexose transport activity of LR67 has been demonstrated in a yeast (*Saccharomyces cerevisiae*) mutant lacking the glucose uptake ability [62, 66]. A preliminary analysis of five protein sequences encoded by *Lr67* [62] including three homoeologs (in A, B, D chromosomes) and the resistance/susceptibility versions from GenBank (accessions: ALL26327, ALL26328, ALL26329, ALL26330 and ALL26331) showed that none of them exhibited a high level of homology to the three family proteins (ABC, NLR and START) analyzed in this study (E-value >1 × 10^−5^ and sequence identity <36%). Since LR67 belongs to the sugar transporter protein (STP) family, a genome-wide search of the STP proteins using the major facilitator, sugar transport domain (InterPro accession IPR005828, a domain present in all the above five LR67 proteins), allowed for discovering 261 STP proteins (E-value <1 × 10^−5^) in wheat. This STP data set along with the ABC and other possible transporter families can be further examined in future studies for their physical relationships with QTLs for partial, adult resistance to multiple pathogens in wheat and other cereals.

This study has focused on annotating and characterizing the physical relationships between *ABC*, *NLR* and *START* genes and QTLs for resistance to leaf rust. Similar efforts can be made for stem and stripe (yellow) rusts. At the time of completion of this work as of February 2017, there is only a limited amount of QTL data available for stem and stripe rust resistance from the T3 database [37] that can be used for such annotation and characterization; in particular, imbalanced availability of QTLs for seedling and adult resistance makes a valid comparative assessment more difficult. As more QTL data become available for stem and stripe rusts, it is conceivable that a similar pattern of the physical relationships between the gene families and QTLs will be identified, just like what we observed in leaf rust.

Our sequence analyses were greatly affected by the fragmentary status of the draft wheat genome assembly [3]. In the Ensembl release 31 of the wheat genome used in this study, the 21 wheat chromosomes were represented by a total of 317,977 assembled scaffold sequences. The average scaffold length was about 20,389 bp (S.D. ± 222 7384 bp), with the median of 3800 bp and maximum of 774,434,471 bp. Because of similar concerns about assembly quality, we chose not to use Ensembl release 33 (the latest release in October 2016), which incorporated short RNA-seq reads and full-length cDNA sequences [42] but seems more fragmented than release 31 according to our assessment. For example, the wheat genome assembly in release 33 consists of 735,943 supercontig sequences (compared with 317,977 in release 31), with an average supercontig length of 18,245 bp (S.D. ± 41,822 bp), median of 2431 bp and maximum of 823,974 bp. The fragmentary assembly of the wheat genome might also have affected our BLAST searches using the primer sequences against the wheat genomic sequences, and this may be the primary reason of why only seven *ABC*, *NLR* and *START* genes were identified within 100Kb to the markers of QTLs for rust resistance (Table 4, Additional File 7). Other studies based on similar fragmentary assemblies were not even able to identify candidate genes but rather some genomic regions for disease resistance and other agronomic traits. Two such studies are: (i) an in silico mapping of DArT marker sequences for identification of genomic regions harboring resistance to the three rusts [67] and (ii) flanking sequences of the SNPs of the wheat 90 K chip for identification of candidate genes and regions for pre-harvest sprouting (PHS) resistance in wheat [68], but with the candidate genes being inferred from Brachypodium (*Brachypodium distachyon*) and rice genomes via comparative mapping.

In addition to the *ABC*, *NLR* and *START* genes analyzed in this study, genes in other families may also be involved in rust resistance. We have already mentioned genes within the STP transporter family like *Lr67*. Additional genes include those encoding unusual kinases, receptor-like kinases (RLKs) and receptor-like proteins (RLPs) [69], and genes involving cell wall biosynthesis and metabolism, such as cysteine proteinase, phenylalanine ammonia-lyase, plasma membrane ATPase and chalcone synthase [58]. When a pathogen attacks its host, the first line of host defense is through secretion of cell surface pattern-recognition receptors (PRRs) and such defense-induced proteins may be recognized and overcome by specific pathogen effectors. The majority of characterized PRRs are either RLKs or RLPs [70–72]. A recently developed tool called RGAugury [73] focused on classification of these proteins and NLR protein family. However, our study had a different focus on the three major protein families related to leaf rust resistance in wheat and these families contain different domains suitable for large-scale analyses.

Certainly with ongoing international efforts for more in-depth wheat genome sequencing, the candidate genes we identified for each of the three gene families eventually will be physically mapped at accurate locations on individual chromosomes. In other words, Fig. 3 will be significantly clarified and updated. Such updating will enable wheat breeders and pathologists to use the well-characterized *ABC*, *NLR* and *START* genes for molecular characterization of the designated *Lr* genes in breeding for rust resistances. While *ABC* and *START* genes may be preferably used as reasoned above, their resistance is only partial and insufficient under high disease pressure when used alone. Strong, durable resistance has been achieved by combining these race-nonspecific genes with race-specific *NLR* genes such as ‘*Sr*2-complex’ [74]. However, little is known about the other combining genes and their interactions in the complex. Thus, it would be desirable that future studies will investigate how race-nonspecific genes in the *ABC* or *START* gene family interact with weak and strong *R* genes in the *NLR* gene family at the molecular level using the candidate genes we identified and characterized for the three gene families.

## Conclusions

In this study we predicted the putative *ABC*, *NLR* and *START* genes in the hexaploid wheat genome, and performed an integrated analysis with the available genetic resources related to rust resistance, including mapped *Lr* genes, SNPs and other molecular markers. Our analysis suggests that the *ABC* (and *START*) genes are more likely to share genomic regions with QTLs for race-nonspecific, adult resistance whereas the *NLR* genes are more likely to share genomic regions with QTLs for race-specific resistance that would be often expressed at the seedling stage. Candidate genes such as those containing one or multiple missense coding SNPs can be tested in future studies. With continuing efforts for producing an improved assembly and annotation of the wheat genome, bioinformatics analyses such as ours can help identify novel genes for rust resistance breeding in wheat.

## Methods

### Prediction of ABC, NLR and START genes in wheat genome

The wheat proteome (release 31) was downloaded from the FTP archive site (ftp://ftp.ensemblgenomes.org/pub/release-31/plants/fasta/triticum_aestivum/pep/Triticum_aestivum.IWGSC1+popseq.31.pep.all.fa) of Ensembl Plants [49]. This database contains a total of 100,344 predicted protein sequences. Using these protein sequences, we predicted the domains within each protein using the standalone software InterProScan (version 5.21–60.0, 64-bit) [47], installed on a CentOS (release 7.2) Linux system with 8-core CPU (1600 MHz) and 32 Gb RAM. The InterProscan E-value cutoff was set to 1 × 10^−5^ [47]. From the InterProscan output file, the presence of PF00005 (ATP-binding domain of ABC transporters) from the protein family database Pfam [46] within a protein sequence was used to select candidate ABC proteins. These candidates were then classified into eight families (A, B, C, D, E, F, G and I) based upon their best BLASTP hit against the known ABC proteins in Arabidopsis and *Oryza sativa* (rice), which were collected from the literature [54, 75]. The G family was further divided into Gwbc (White Brown Box) and Gpdr (Pleiotropic Drug Resistance). For the START family, we used the presence of the START domain (InterPro: IPR002913 and IPR005031) or START-like domain (IPR023393) as the signature, and grouped the putative START proteins into five categories: MINIMAL-START, START, HD-START, HD-START-MEKHLA and OTHER. This classification scheme is similar to those reported in previous studies [22, 48]. A MINIMAL-START protein only contains a single START or START-like domain, whereas a START protein contains multiple domains with at least one START or START-like domain. Moreover, a multidomain START protein was designated as HD-START if it contains a homeodomain-like domain (IPR009057) and as HD-START-MEKHLA if it also contains a MEKHLA domain (IPR013978). The NBS-LRR resistance proteins were predicted using the NLR-Parser tool [76] in a two-step process. First, the entire wheat proteome was scanned with the MEME-formatted motifs (meme.xml) using MAST (Motif Alignment and Scanning Tool) in the MEME (version 4.9.1) suite [77]. Second, the MAST output (mast.xml) was parsed and the NBSLRR genes were classified with NLR-Parser into three classes: CNL (COIL-NBS-LRR), TNL (TIR-NBS-LRR) and N/A.

### In silico mapping of the QTL SNP markers to *ABC*, *NLR* and *START* genes on the wheat chromosomes

Two data sets, each with 2500 quantitative trait loci (QTLs) for leaf rust resistance at seedling or adult stage, were extracted from the “GWAS Results” in the Triticeae Toolbox (T3) database (https://triticeaetoolbox.org/wheat/qtl/qtl_report.php) [37]. By selecting ‘Biotic stress’ in the “Category” column and by clicking appropriate leaf rust traits at adult or seedling stage in the “Traits” column, the T3 would display the results from the GWAS analysis for associations between markers (including Infinium 9 K, Infinium 90 K, and GBS restriction sites) and traits for individual trials (individual locations or inoculum types) within the T3 database. The GWAS analysis was carried out using rrBLUP GWAS package [78] for individual trials or the combined analysis across all trials with the genotype-by-environment interaction effect being adjusted by including those principle components that accounted for more than 5% of the environment-relationship matrix variance as fixed effects in the mixed-model analysis. For a better control of false positive rates in these preliminary QTL data sets, we applied a significance level of *P* ≤ 0.01 which allowed for retaining 1090 QTLs in seedlings and 1542 QTLs in adult plants for subsequent analysis (Additional file 4).

The sequences of the SNP markers from the GWAS results described above were obtained in T3 and CerealsDB [79]. To predict their functional consequences, these SNPs were first converted into a VCF (Variant Call Format) and annotated with VEP [80]. Then, to better estimate the genomic position of each SNP and its proximity to the predicted genes in the *ABC*, *NLR* and *START* gene families, we mapped the sequence (removing the ‘/’, B allele and the surrounding square brackets) flanking each SNP by alignment to the wheat genome assembly (Triticum_aestivum.IWGSC1 + popseq.31.dna_sm.toplevel.fa) using BLASTN (version 2.5.0+) in the BLAST+ package [50]. This assembly was generated by Ensembl Plants from the whole genome shotgun sequencing data [2], a chromosome-based draft sequence [3] and the POPSEQ (population sequencing) data [81]. The chromosomal positions of these genes were based on the Ensembl annotation file (Triticum_aestivum.IWGSC1 + popseq.31.gff3) in GFF3 (General Feature Format) format. Both files were downloaded from the Ensembl FTP archive site (ftp://ftp.ensemblgenomes.org/pub/release-31/plants/). As each flanking sequence often matches several genomic regions, we used E-value, alignment identity and query coverage as criteria to retain the top three candidates that usually represent one hit in each of the three wheat genomes.

For the SNPs located in the genomic regions covering the *ABC*, *NLR* and *START* genes, those in the coding regions were recognized through aligning their flanking sequences with their coding sequence (CDS) downloaded from Ensembl. We then compared the two alleles of each SNP marker with the allele at the same position of the Ensembl CDS and determined if the Ensembl allele (as reference) was the same as the A or B allele at the SNP site, or in some cases differed from both alleles. To predict the amino acid change, we replaced the Ensembl CDS with the A or B allele, translated the newly generated CDS into protein sequence using the standard codon table. Therefore, if the Ensembl reference allele matches the A or B allele at the SNP site, two protein sequences were generated and otherwise three protein sequences were translated for each SNP marker. To verify this process, we utilized the known SNP (and deletion) in the *Lr34* CDS as well as the resistant and susceptible LR34 proteins [6, 15]. We then found the amino acid difference at the corresponding position of each protein translated from each CDS containing different SNP alleles.

### Prediction of candidate genes based on sequences of non-SNP markers in wheat

We collected a set of 99 non-SNP markers that showed significant (a LOD score of ≥3.0) associations with wheat rust resistance from Liu et al. [57] and other sources. The primer sequences of these markers were found in GrainGenes [82], MASWheat (http://maswheat.ucdavis.edu/protocols/stemrust/) and the literature (Additional file 7). Similar to the above analysis of SNP flanking sequences, we searched the primer sequences against the wheat genome sequence assembly (Triticum_aestivum.IWGSC1 + popseq.31.dna_sm.toplevel.fa) using BLASTN [50] to identify genomic regions covered by these markers. Each primer query was generated by concatenating the forward primer and reverse primer with five ‘N’ letters inserted between them as gaps. Because of the short length of each primer pair (usually less than 40 bp), blastn-short, a BLASTN program optimized for sequences shorter than 50 bases [50], was used with settings of a word size of seven and a relaxed E-value of 100. To compare chromosomal positions of markers and candidate genes, we also used the Ensembl annotation file (Triticum_aestivum.IWGSC1 + popseq.31.gff3).

### Transcription analysis

Dobon et al. [27] recently published a RNA-seq time-course data set for a resistant line (Avocet-*Yr5*) and a susceptible wheat cultivar (Vuka) inoculated with yellow rust pathogen *Puccinia striiformis* f. sp. tritici (*Pst*). The rust inoculum (isolate 87/66 of *Pst*) is avirulent to the resistant line (Avocet-*Yr5*), but virulent to the susceptible cultivar (Vuka). We analyzed the expression values (Transcripts Per Million reads or TPM) of 123,532 wheat transcripts (based on the annotation of Ensembl Plants [49]) taken from Supplemental Tables S20 and S21 of Dobon et al. [27]. We identified putative *ABC*, *NLR* and *START* genes from those host transcripts and analyzed their expression profiles at different days post inoculation (dpi) in the resistant and susceptible wheat plants. The original data on the expression profiles contain five time points (0, 1, 2, 3, and 5 dpi) for Avocet-*Yr5*, and eight (0, 1, 2, 3, 5, 7, 9, and 11 dpi) for Vuka. We then extracted the TPM values for the putative *ABC*, *NLR* and *START* genes, eliminating those with zero values on all dpi. For each of the putative genes in the three gene families, we calculated a fold change as the sum of the five expression values in the resistant host divided by the sum in the susceptible host. In addition, we identified *ABC*, *NLR* and *START* genes expressed exclusively in the resistant host or the susceptible host.

## Additional files

Additional file 1: Summary of the family classification of the ABC, NLR and START proteins in wheat and their predicted InterPro domains as well as the description for each InterPro identifier. (XLSX 141 kb)

Additional file 2: Annotation of the putative *ABC*, *NLR* and *START* genes in wheat using the sequences of plant resistance genes in GenBank and PRDdb. (XLSX 228 kb)

Additional file 3: Table S1. Summary of the ABC transporter family, domain structures and major functions in plants. **Table S2.** The VEP annotation of the 1809 unique SNPs associated with leaf rust resistance in seedling and adult wheat plants from the T3 database. **Figure S1.** The Venn diagram (A) of the SNP markers of leaf rust resistance QTLs in seedlings and adult plants in the T3 database and their distribution on different wheat chromosomes (B: seedlings; C: adult plants). (PDF 574 kb)

Additional file 4: The leaf rust resistance QTLs (*P* ≤ 0.01) from the T3 database of the wheat seedlings and adult plants and the flanking sequences of their SNP markers from CerealsDB and T3 database. (XLSX 335 kb)

Additional file 5: The SNP markers located within (marker located between the gene start and the gene end on the same chromosome; highlighted) ABC, NLR and START genes and 5 Kb upstream (from transcription start site or TSS) or downstream (from transcription termination site or TTS) of them. (XLSX 78 kb)

Additional file 6: Predicted effects of rust SNPs on the amino acid changes in the proteins encoded by the putative ABC, NLR and START genes. Genes with multiple SNPs were highlighted. (XLSX 50 kb)

Additional file 7: The molecular markers and their primer sequences found in GrainGenes and candidate genes near these makers. (XLSX 22 kb)

Additional file 8: RNA-seq expression values (in unit of TPM or transcripts per million) of *ABC*, *NLR* and *START* genes in resistant and susceptible wheat hosts at five time points (0 dpi, 1 dpi, 2 dpi, 3 dpi and 5 dpi or days post inoculation), fold changes of genes expressed in both resistant and susceptible wheat, and genes expressed only in the resistant or susceptible wheat. (XLSX 211 kb)

## Abbreviations

ABC: ATP-binding cassette
CDS: coding sequence
DPI: days post inoculation
NLR: nucleotide-binding site /leucine-rich repeat (NBS-LRR)
Pdr: pleiotropic drug resistance
QTL: quantitative trait locus
SNP: single nucleotide polymorphism
START: steroidogenic acute regulatory (StAR) protein-related lipid transfer domain
STP: sugar transporter
UTR: untranslated region
Wbc: white brown complex

## References

[1] Choulet F, Alberti A, Theil S, Glover N, Barbe V, Daron J, Pingault L, Sourdille P, Couloux A, Paux E (2014). Structural and functional partitioning of bread wheat chromosome 3B. Science.

[2] Brenchley R, Spannagl M, Pfeifer M, Barker GLA, D'more R, Allen AM, McKenzie N, Kramer M, Kerhornou A, Bolser D (2012). Analysis of the bread wheat genome using whole-genome shotgun sequencing. Nature.

[3] The International Wheat Genome Sequencing Consortium (2014). A chromosome-based draft sequence of the hexaploid bread wheat (*Triticum aestivum*) genome. Science.

[4] McIntosh R, Dubcovsky J, Rogers W, Morris C, Appels R, Xia X, AZUL B (2013). Catalogue of gene symbols for wheat: 2013–2014.

[5] Kolmer JA, Singh RP, Garvin DF, Viccars L, William HM, Huerta-Espino J, Ogbonnaya FC, Raman H, Orford S, Bariana HS (2008). Analysis of the Lr34/Yr18 rust resistance region in wheat germplasm. Crop Sci.

[6] Krattinger SG, Lagudah ES, Spielmeyer W, Singh RP, Huerta-Espino J, McFadden H, Bossolini E, Selter LL, Keller B (2009). A putative ABC transporter confers durable resistance to multiple fungal pathogens in wheat. Science.

[7] Cloutier S, McCallum BD, Loutre C, Banks TW, Wicker T, Feuillet C, Keller B, Jordan MC (2007). Leaf rust resistance gene Lr1, isolated from bread wheat (*Triticum aestivum* L.) is a member of the large psr567 gene family. Plant Mol Biol.

[8] Feuillet C, Travella S, Stein N, Albar L, Nublat A, Keller B (2003). Map-based isolation of the leaf rust disease resistance gene Lr10 from the hexaploid wheat (*Triticum aestivum* L.) genome. Proc Natl Acad Sci U S A.

[9] Huang L, Brooks SA, Li WL, Fellers JP, Trick HN, Gill BS (2003). Map-based cloning of leaf rust resistance gene Lr21 from the large and polyploid genome of bread wheat. Genetics.

[10] Krattinger SG, Sucher J, Selter LL, Chauhan H, Zhou B, Tang MZ, Upadhyaya NM, Mieulet D, Guiderdoni E, Weidenbach D (2016). The wheat durable, multipathogen resistance gene Lr34 confers partial blast resistance in rice. Plant Biotechnol J.

[11] Dyck PL (1987). The association of a gene for leaf rust resistance with the chromosome 7D suppressor of stem rust resistance in common wheat. Genome.

[12] Risk JM, Selter LL, Chauhan H, Krattinger SG, Kumlehn J, Hensel G, Viccars LA, Richardson TM, Buesing G, Troller A (2013). The wheat Lr34 gene provides resistance against multiple fungal pathogens in barley. Plant Biotechnol J.

[13] Lagudah ES, Krattinger SG, Herrera-Foessel S, Singh RP, Huerta-Espino J, Spielmeyer W, Brown-Guedira G, Selter LL, Keller B (2009). Gene-specific markers for the wheat gene Lr34/Yr18/Pm38 which confers resistance to multiple fungal pathogens. Theor Appl Genet.

[14] Spielmeyer W, McIntosh RA, Kolmer J, Lagudah ES (2005). Powdery mildew resistance and Lr34/Yr18 genes for durable resistance to leaf and stripe rust cosegregate at a locus on the short arm of chromosome 7D of wheat. Theor Appl Genet.

[15] Krattinger SG, Lagudah ES, Wicker T, Risk JM, Ashton AR, Selter LL, Matsumoto T, Keller B (2011). Lr34 multi-pathogen resistance ABC transporter: molecular analysis of homoeologous and orthologous genes in hexaploid wheat and other grass species. Plant J.

[16] Fu DL, Uauy C, Distelfeld A, Blechl A, Epstein L, Chen XM, Sela HA, Fahima T, Dubcovsky J (2009). A kinase-START Gene confers temperature-dependent resistance to wheat stripe rust. Science.

[17] Fukuoka S, Saka N, Koga H, Ono K, Shimizu T, Ebana K, Hayashi N, Takahashi A, Hirochika H, Okuno K (2009). Loss of function of a proline-containing protein confers durable disease resistance in Rice. Science.

[18] Buschges R, Hollricher K, Panstruga R, Simons G, Wolter M (1997). Frijters A, vanDaelen R, vanderLee T, Diergaarde P, Groenendijk J *et al*: The barley mlo gene: A novel control element of plant pathogen resistance. Cell.

[19] Kim J, Lim CJ, Lee BW, Choi JP, Oh SK, Ahmad R, Kwon SY, Ahn J, Hur CG (2012). A genome-wide comparison of NB-LRR type of resistance gene analogs (RGA) in the plant kingdom. Mol Cells.

[20] van Ooijen G, van den Burg HA, Cornelissen BJC, Takken FLW (2007). Structure and function of resistance proteins in solanaceous plants. Annu Rev Phytopathol.

[21] Kang J, Park J, Choi H, Burla B, Kretzschmar T, Lee Y, Martinoia E (2011). Plant ABC transporters. The Arabidopsis Book / American Society of Plant Biologists.

[22] Satheesh V, Chidambaranathan P, Jagannadham PT, Kumar V, Jain PK, Chinnusamy V, Bhat SR, Srinivasan R: Transmembrane START domain proteins: in silico identification, characterization and expression analysis under stress conditions in chickpea (*Cicer arietinum* L.). *Plant Signal Behav* 2016, 11(2):e992698. 10.4161/15592324.2014.992698 PMC4883873 26445326

[23] Schrick K, Bruno M, Khosla A, Cox PN, Marlatt SA, Roque RA, Nguyen HC, He C, Snyder MP, Singh D (2014). Shared functions of plant and mammalian StAR-related lipid transfer (START) domains in modulating transcription factor activity. BMC Biol.

[24] Wang Z, Gerstein M, Snyder M (2009). RNA-Seq: a revolutionary tool for transcriptomics. Nat Rev Genet.

[25] Xu GR, Strong MJ, Lacey MR, Baribault C, Flemington EK, Taylor CM: RNA CoMPASS: A Dual Approach for Pathogen and Host Transcriptome Analysis of RNA-Seq Datasets. PLoS One 2014, **9**(2). 10.1371/journal.pone.0089445 PMC3934900 24586784

[26] Westermann AJ, Gorski SA, Vogel J (2012). Dual RNA-seq of pathogen and host. Nat Rev Microbiol.

[27] Dobon A, Bunting DCE, Cabrera-Quio LE, Uauy C, Saunders DGO (2016). The host-pathogen interaction between wheat and yellow rust induces temporally coordinated waves of gene expression. BMC Genomics.

[28] Soriano JM, Royo C (2015). Dissecting the genetic architecture of leaf rust resistance in wheat by QTL meta-analysis. Phytopathology.

[29] Bajgain P, Rouse MN, Bhavani S, Anderson JA (2015). QTL mapping of adult plant resistance to Ug99 stem rust in the spring wheat population RB07/MN06113-8. Mol Breed.

[30] Rosewarne GM, Herrera-Foessel SA, Singh RP, Huerta-Espino J, Lan CX, He ZH (2013). Quantitative trait loci of stripe rust resistance in wheat. Theor Appl Genet.

[31] Njau PN, Bhavani S, Huerta-Espino J, Keller B, Singh RP (2013). Identification of QTL associated with durable adult plant resistance to stem rust race Ug99 in wheat cultivar 'Pavon 76′. Euphytica.

[32] Zegeye H, Rasheed A, Makdis F, Badebo A, Ogbonnaya FC (2014). Genome-wide association mapping for seedling and adult plant resistance to stripe rust in synthetic Hexaploid wheat. PLoS One.

[33] Gao LL, Turner MK, Chao SM, Kolmer J, Anderson JA (2016). Genome wide association study of seedling and adult plant leaf rust resistance in elite spring wheat breeding lines. PLoS One.

[34] Maccaferri M, Zhang JL, Bulli P, Abate Z, Chao SM, Cantu D, Bossolini E, Chen XM, Pumphrey M, Dubcovsky J: A Genome-Wide Association Study of Resistance to Stripe Rust (Puccinia striiformis f. sp tritici) in a Worldwide Collection of Hexaploid Spring Wheat (*Triticum aestivum* L.). *G3-Genes Genom Genet* 2015, **5**(3):449–465. 10.1534/g3.114.014563 PMC4349098 25609748

[35] Cavanagh CR, Chao S, Wang S, Huang BE, Stephen S, Kiani S, Forrest K, Saintenac C, Brown-Guedira GL, Akhunova A (2013). Genome-wide comparative diversity uncovers multiple targets of selection for improvement in hexaploid wheat landraces and cultivars. Proc Natl Acad Sci U S A.

[36] Wang SC, Wong DB, Forrest K, Allen A, Chao SM, Huang BE, Maccaferri M, Salvi S, Milner SG, Cattivelli L (2014). Characterization of polyploid wheat genomic diversity using a high-density 90 000 single nucleotide polymorphism array. Plant Biotechnol J.

[37] Blake VC, Birkett C, Matthews DE, Hane DL, Bradbury P, Jannink JL, et al., 2. Plant Genome-Us. 2016:9(2). 10.3835/plantgenome2014.12.0099 27898834

[38] Crouzet J, Trombik T, Fraysse AS, Boutry M (2006). Organization and function of the plant pleiotropic drug resistance ABC transporter family. FEBS Lett.

[39] Andolfo G, Ruocco M, Di Donato A, Frusciante L, Lorito M, Scala F, et al. Genetic variability and evolutionary diversification of membrane ABC transporters in plants. BMC Plant Biol. 2015;15 10.1186/s12870-014-0323-2 PMC4358917 25850033

[40] Sekhwal MK, Li P, Lam I, Wang X, Cloutier S, You FM (2015). Disease resistance Gene analogs (RGAs) in plants. Int J Mol Sci.

[41] Bouktila D, Khalfallah Y, Habachi-Houimli Y, Mezghani-Khemakhem M, Makni M, Makni H (2014). Large-scale analysis of NBS domain-encoding resistance gene analogs in Triticeae. Genet Mol Biol.

[42] Clavijo BJ, Venturini L, Schudoma C, Accinelli GG, Kaithakottil G, Wright J, Borrill P, Kettleborough G, Heavens D, Chapman H (2017). An improved assembly and annotation of the allohexaploid wheat genome identifies complete families of agronomic genes and provides genomic evidence for chromosomal translocations. Genome Res.

[43] Yu J, Tehrim S, Zhang F, Tong C, Huang J, Cheng X, Dong C, Zhou Y, Qin R, Hua W (2014). Genome-wide comparative analysis of NBS-encoding genes between brassica species and *Arabidopsis thaliana*. BMC Genomics.

[44] Meyers BC, Kozik A, Griego A, Kuang HH, Michelmore RW (2003). Genome-wide analysis of NBS-LRR-encoding genes in Arabidopsis. Plant Cell.

[45] Tarr DEK, Alexander HM (2009). TIR-NBS-LRR genes are rare in monocots: evidence from diverse monocot orders. BMC Res Notes.

[46] Finn RD, Coggill P, Eberhardt RY, Eddy SR, Mistry J, Mitchell AL, Potter SC, Punta M, Qureshi M, Sangrador-Vegas A (2016). The Pfam protein families database: towards a more sustainable future. Nucleic Acids Res.

[47] Jones P, Binns D, Chang HY, Fraser M, Li WZ, McAnulla C, McWilliam H, Maslen J, Mitchell A, Nuka G (2014). InterProScan 5: genome-scale protein function classification. Bioinformatics.

[48] Schrick K, Nguyen D, Karlowski WM, Mayer KFX (2004). START lipid/sterol-binding domains are amplified in plants and are predominantly associated with homeodomain transcription factors. Genome Biol.

[49] Cunningham F, Amode MR, Barrell D, Beal K, Billis K, Brent S, Carvalho-Silva D, Clapham P, Coates G, Fitzgerald S (2015). Ensembl 2015. Nucleic Acids Res.

[50] Camacho C, Coulouris G, Avagyan V, Ma N, Papadopoulos J, Bealer K, Madden TL (2009). BLAST plus : architecture and applications. BMC Bioinformatics.

[51] Clark K, Karsch-Mizrachi I, Lipman DJ, Ostell J, Sayers EW (2016). GenBank. Nucleic Acids Res.

[52] Sanseverino W, Hermoso A, D'Alessandro R, Vlasova A, Andolfo G, Frusciante L, Lowy E, Roma G, Ercolano MR (2013). PRGdb 2.0: towards a community-based database model for the analysis of R-genes in plants. Nucleic Acids Res.

[53] Rea PA (2007). Plant ATP-binding cassette transporters. Annu Rev Plant Biol.

[54] Verrier PJ, Bird D, Buria B, Dassa E, Forestier C, Geisler M, Klein M, Kolukisaoglu U, Lee Y, Martinoia E (2008). Plant ABC proteins - a unified nomenclature and updated inventory. Trends Plant Sci.

[55] Kretzschmar T, Burla B, Lee Y, Martinoia E, Nagy R (2011). Functions of ABC transporters in plants. Essays Biochem.

[56] Basnet BR, Singh S, Lopez-Vera EE, Huerta-Espino J, Bhavani S, Jin Y, Rouse MN, Singh RP (2015). Molecular mapping and validation of SrND643: a new wheat Gene for resistance to the stem rust pathogen Ug99 race group. Phytopathology.

[57] Liu SY, Rudd JC, Bai GH, Haley SD, Ibrahim AMH, Xue QW, Hays DB, Graybosch RA, Devkota RN, St Amand P (2014). Molecular markers linked to important genes in hard winter wheat. Crop Sci.

[58] Manickavelu A, Kawaura K, Oishi K, Shin IT, Kohara Y, Yahiaoui N, Keller B, Suzuki A, Yano K, Ogihara Y (2010). Comparative gene expression analysis of susceptible and resistant near-isogenic lines in common wheat infected by Puccinia triticina. DNA Res.

[59] Dmochowska-Boguta M, Alaba S, Yanushevska Y, Piechota U, Lasota E, Nadolska-Orczyk A, Karlowski WM, Orczyk W (2015). Pathogen-regulated genes in wheat isogenic lines differing in resistance to brown rust Puccinia triticina. BMC Genomics.

[60] Hulbert SH, Bai J, Fellers JP, Pacheco MG, Bowden RL (2007). Gene expression patterns in near isogenic lines for wheat rust resistance gene Lr34/Yr18. Phytopathology.

[61] Moore JW, Herrera-Foessel S, Lan C, Schnippenkoetter W, Ayliffe M, Huerta-Espino J, Lillemo M, Viccars L, Milne R, Periyannan S (2015). A recently evolved hexose transporter variant confers resistance to multiple pathogens in wheat. Nat Genet.

[62] Zmienko A, Samelak A, Kozlowski P, Figlerowicz M (2014). Copy number polymorphism in plant genomes. Theor Appl Genet.

[63] Moore G (1995). Cereal genome evolution - pastoral pursuits with Lego genomes. Curr Opin Genet Dev.

[64] Herrera-Foessel SA, Singh RP, Huerta-Espino J, Rosewarne GM, Periyannan SK, Viccars L, Calvo-Salazar V, Lan CX, Lagudah ES (2012). Lr68: a new gene conferring slow rusting resistance to leaf rust in wheat. Theor Appl Genet.

[65] Singla J, Luthi L, Wicker T, Bansal U, Krattinger SG, Keller B (2017). Characterization of Lr75: a partial, broad-spectrum leaf rust resistance gene in wheat. Theor Appl Genet.

[66] White FF, Frommer W (2015). Deciphering durable resistance one R gene at a time. Nat Genet.

[67] Li HH, Singh S, Bhavani S, Singh RP, Sehgal D, Basnet BR, Vikram P, Burgueno-Ferreira J, Huerta-Espino J (2016). Identification of genomic associations for adult plant resistance in the background of popular south Asian wheat cultivar, PBW343. Front Plant Sci.

[68] Cabral AL, Jordan MC, McCartney CA, You FM, Humphreys DG, MacLachlan R, Pozniak CJ (2014). Identification of candidate genes, regions and markers for pre-harvest sprouting resistance in wheat (*Triticum aestivum* L.). BMC Plant Biol.

[69] Krattinger SG, Keller B (2016). Molecular genetics and evolution of disease resistance in cereals. New Phytol.

[70] Zipfel C (2014). Plant pattern-recognition receptors. Trends Immunol.

[71] Monaghan J, Zipfel C (2012). Plant pattern recognition receptor complexes at the plasma membrane. Curr Opin Plant Biol.

[72] Bohm H, Albert I, Fan L, Reinhard A, Nurnberger T (2014). Immune receptor complexes at the plant cell surface. Curr Opin Plant Biol.

[73] Li PC, Quan XD, Jia GF, Xiao J, Cloutier S, You FM (2016). RGAugury: a pipeline for genome-wide prediction of resistance gene analogs (RGAs) in plants. BMC Genomics.

[74] Singh RP, Hodson DP, Jin Y, Lagudah ES, Ayliffe MA, Bhavani S, Rouse MN, Pretorius ZA, Szabo LJ, Huerta-Espino J (2015). Emergence and spread of new races of wheat stem rust fungus: continued threat to food security and prospects of genetic control. Phytopathology.

[75] Andolfo G, Ruocco M, Di Donato A, Frusciante L, Lorito M, Scala F, Ercolano MR (2015). Genetic variability and evolutionary diversification of membrane ABC transporters in plants. BMC Plant Biol.

[76] Steuernagel B, Jupe F, Witek K, Jones JDG, Wulff BBH (2015). NLR-parser: rapid annotation of plant NLR complements. Bioinformatics.

[77] Bailey TL, Boden M, Buske FA, Frith M, Grant CE, Clementi L, Ren JY, Li WW, Noble WS (2009). MEME SUITE: tools for motif discovery and searching. Nucleic Acids Res.

[78] Endelman JB (2011). Ridge regression and other kernels for genomic selection with R package rrBLUP. Plant Genome-Us.

[79] Wilkinson PA, Winfield MO, Barker GLA, Tyrrell S, Bian X, Allen AM, Burridge A, Coghill JA, Waterfall C, Caccamo M (2016). CerealsDB 3.0: expansion of resources and data integration. BMC Bioinformatics.

[80] McLaren W, Gil L, Hunt SE, Riat HS, Ritchie GRS, Thormann A, Flicek P, Cunningham F (2016). The Ensembl variant effect predictor. Genome Biol.

[81] Chapman JA, Mascher M, Buluc A, Barry K, Georganas E, Session A, Strnadova V, Jenkins J, Sehgal S, Oliker L (2015). A whole-genome shotgun approach for assembling and anchoring the hexaploid bread wheat genome. Genome Biol.

[82] Carollo V, Matthews DE, Lazo GR, Blake TK, Hummel DD, Lui N, Hane DL, Anderson OD (2005). GrainGenes 2.0. An improved resource for the small-grains community. Plant Physiol.

