# Supplementary material for: Prediction and analysis of three gene families related to leaf rust (Puccinia triticina) resistance in wheat (Triticum aestivum L.)
Source: BMC Plant Biol. 2017 Jun 20;17:108. doi: 10.1186/s12870-017-1056-9 (PMC5477749; doi:10.1186/s12870-017-1056-9)

Table S1. The eight families of the plant ABC transporter superfamily, their domain organization and major functions in Arabidopsis

Family	Domain organization	Feature	Major function
A	$(TMD\text{-}NBD)_2$ or TMD-NBD	Full-size or half size; forward orientation	Lipid transport
B	$(TMD\text{-}NBD)_2$ or TMD-NBD	Full-size or half size; forward orientation	Transport of auxin, secondary metabolites and xenobiotics
C	$(TMD\text{-}NBD)_2$	Full-size; forward orientation	Phytate transport, xenobiotic transport, folate storage, vacuolar compartmentation of anthocyanin
D	$(TMD\text{-}NBD)_2$ or TMD-NBD	Full-size or half size; forward orientation	Fatty acid beta-oxidation, fatty-acyl-CoA transport, IBA (indole-3-butyric acid) transport, peroxisomal import of fatty acids
E	NBD-NBD	Soluble (no membrane-bound TMD)	Ribosomal subunit export from nucleus, translational initiation/termination, suppression of RNA silencing
F	NBD-NBD	Soluble (no membrane-bound TMD)	Ribosome recycling and translational control, defense response to bacterium, response to cadmium ion
G	NBD-TMD	Gwbc group; half size; reverse orientation	Kanamycin resistance, transmembrane transport, jasmonate response, salicylic acid response
	$(NBD\text{-}TMD)_2$	Gpdr group; full size; reverse orientation; plant/fungus specific	Pathogen defense response, cuticle formation, drug transmembrane transport, organic hydroxy compound transport, p-coumaryl alcohol transport, regulation of lignin biosynthesis
I	TMD or NBD or CYT or SBP	Only one ABC-related domain, similar to prokaryotic ABC transporters	Cytochrome complex assembly, heme transport, protein complex assembly, respiratory chain complex IV assembly

Abbreviations: TMD: transmembrane domain, NBD: nucleotide-binding domain (ATP-binding cassette), CYT: conserved soluble protein which interacts with ABC domain, SSA: substrate binding protein, WBC: white-brown complex, PDR: pleiotropic drug resistance

Table S2. The VEP annotation of SNPs used for leaf rust resistance association studies linked to the genes in the ABC, NLR and START families and other genomic regions in wheat

	ABC	NLR	START	OTHERS[1]
Coding	5	6	1	714
Stop codon[2]	0	0	0	27
Intron[3]	2	0	0	114
5'UTR	0	0	0	26
3'UTR	3	2	3	210
Upstream	1	4	0	222
Downstream	2	1	2	356
Intergenic		174		

[1] SNPs were annotated within or 5 Kb upstream or downstream of the genes not in the ABC, NLR and START families.[2] SNPs affect stop codons including gain or loss. [3] SNPs are located in an intron including splice sites such as splice acceptor or donor

Figure S1. The Venn diagram (A) of the SNP markers of leaf rust resistance QTLs ($P \leq 0.01$) for seedling and adult stages downloaded from the T3 database and their distribution on different wheat chromosomes (B: seedling; C: adult)

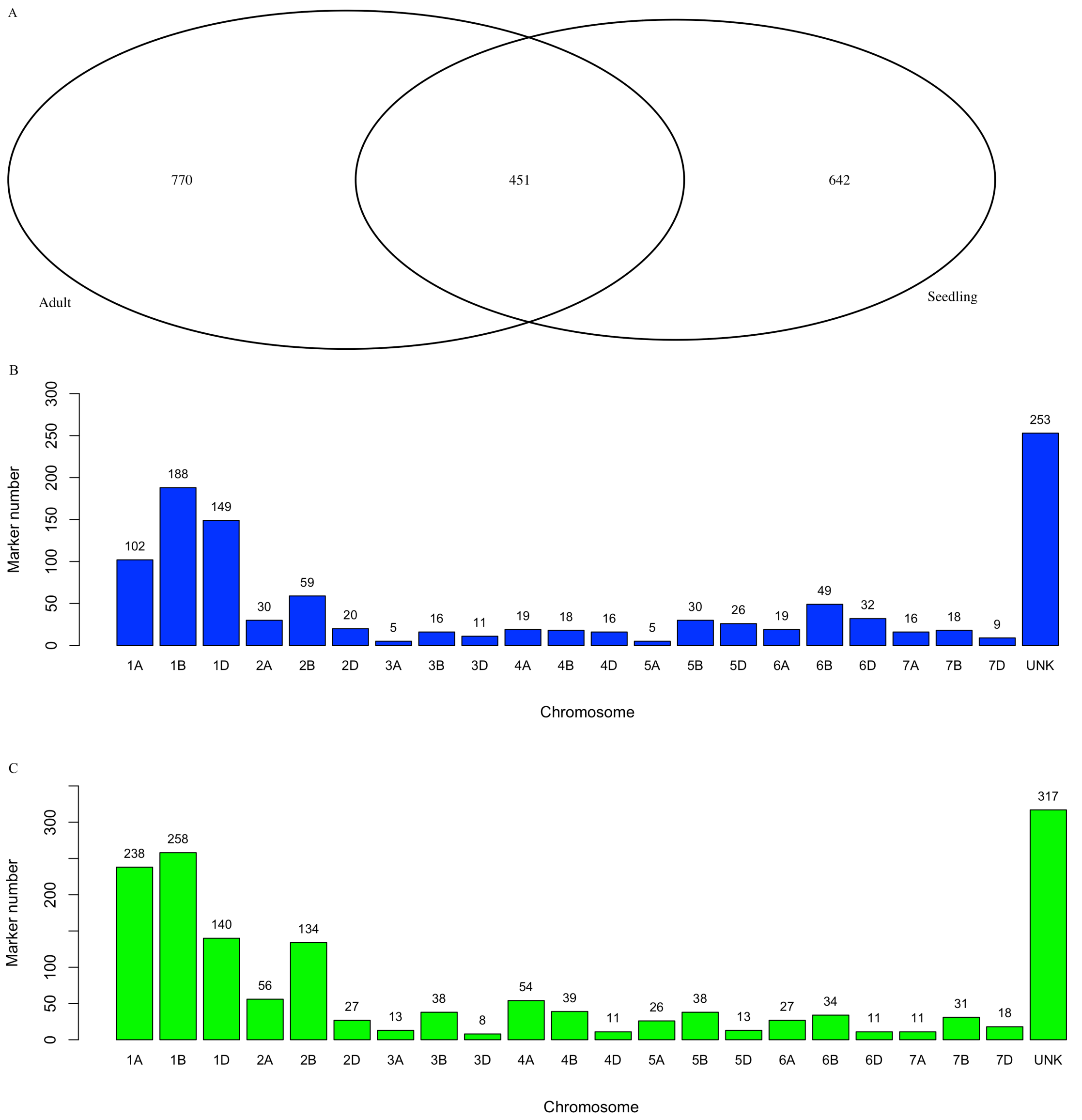

Supplement: Supplementary file 3 — Summary of the ABC transporter family, domain structures and major functions in plants. Table S2. The VEP annotation of the 1809 unique SNPs associated with leaf rust resistance in seedling and adult wheat plants from the T3 database. Figure S1. The Venn diagram (A) of the SNP markers of leaf rust resistance QTLs in seedlings and adult plants in the T3 database and their distribution on different wheat chromosomes (B: seedlings; C: adult plants). (PDF 574 kb) [file 12870_2017_1056_MOESM3_ESM.pdf]
